# Casein kinase II phosphorylation of cyclin F at serine 621 regulates the Lys48-ubiquitylation E3 ligase activity of the SCF^(cyclin F)^ complex

**DOI:** 10.1098/rsob.170058

**Published:** 2017-10-11

**Authors:** Albert Lee, Stephanie L. Rayner, Alana De Luca, Serene S. L. Gwee, Marco Morsch, Vinod Sundaramoorthy, Hamideh Shahheydari, Audrey Ragagnin, Bingyang Shi, Shu Yang, Kelly L. Williams, Emily K. Don, Adam K. Walker, Katharine Y. Zhang, Justin J. Yerbury, Nicholas J. Cole, Julie D. Atkin, Ian P. Blair, Mark P. Molloy, Roger S. Chung

**Affiliations:** 1 Department of Biomedical Sciences, Centre for Motor Neuron Disease Research, Faculty of Medicine and Health Sciences, Macquarie University, 2 Technology Place, North Ryde, NSW 2109, Australia; 2 Australian Proteome Analysis Facility, Research Park Drive, Macquarie University, North Ryde, NSW 2109, Australia; 3 Faculty of Science and Engineering, Department of Chemistry and Biomolecular Sciences, Research Park Drive, Macquarie University, North Ryde, NSW 2109, Australia; 4 Illawarra Health and Medical Research Institute, School of Biological Sciences, University of Wollongong, Northfields Avenue, Wollongong, NSW 2522, Australia; 5 Department of Biochemistry and Genetics, La Trobe Institute for Molecular Science, Victoria, Australia

**Keywords:** ubiquitylation, phosphorylation, CCNF, cyclin F, amyotrophic lateral sclerosis, frontotemporal dementia

## Abstract

Amyotrophic lateral sclerosis (ALS) is a fatal neurodegenerative disorder that is characterized by progressive weakness, paralysis and muscle loss often resulting in patient death within 3–5 years of diagnosis. Recently, we identified disease-linked mutations in the *CCNF* gene, which encodes the cyclin F protein, in cohorts of patients with familial and sporadic ALS and frontotemporal dementia (FTD) (Williams KL *et al*. 2016 *Nat. Commun.* **7**, 11253. (doi:10.1038/ncomms11253)). Cyclin F is a part of a Skp1-Cul-F-box (SCF) E3 ubiquitin-protein ligase complex and is responsible for ubiquitylating proteins for degradation by the proteasome. In this study, we investigated the phosphorylation status of cyclin F and the effect of the serine to glycine substitution at site 621 (S621G) on E3 ligase activity. This specific mutation (S621G) was found in a multi-generational Australian family with ALS/FTD. We identified seven phosphorylation sites on cyclin F, of which five are newly reported including Ser621. These phosphorylation sites were mostly identified within the PEST (proline, glutamic acid, serine and threonine) sequence located at the C-terminus of cyclin F. Additionally, we determined that casein kinase II (CK2) can phosphorylate Ser621 and thereby regulate the E3 ligase activity of the SCF^(cyclin F)^ complex. Furthermore, the S621G mutation in cyclin F prevents phosphorylation by CK2 and confers elevated Lys48-ubiquitylation activity, a hallmark of ALS/FTD pathology. These findings highlight the importance of phosphorylation in regulating the activity of the SCF^(cyclin F)^ E3 ligase complex that can affect downstream processes and may lead to defective motor neuron development, neuron degeneration and ultimately ALS and FTD.

## 1. Introduction

Amyotrophic lateral sclerosis (ALS) and frontotemporal dementia (FTD) are fatal neurodegenerative disorders that have common and convergent molecular and pathogenic features [1]. ALS is characterized by progressive weakness, paralysis and muscle loss often resulting in patient death within 3–5 years of diagnosis [2]. Many studies of the biological mechanisms of ALS and FTD have used *in vivo* and *in vitro* models with genetic mutations found in familial cases to recapitulate features of the disease. Around 20% of ALS patients show signs of frontotemporal dementia (FTD) and segregation of both ALS and FTD may be seen within individual families, particularly those with mutations in *C9ORF72* [3,4]. Familial ALS cases appear clinically indistinguishable from sporadic cases [5], and disease models that incorporate familial gene mutations offer an opportunity to better understand the biology of both familial and sporadic ALS. Genetic analyses of ALS-affected families have identified mutations in many genes, including *SOD1, TARDBP, FUS, UBQLN2, VCP, OPTN* and *C9ORF72* [6–13]. The proteins encoded by these genes generally cluster into two major functional groups: RNA/DNA synthesis and processing (e.g. TDP-43 and FUS), and protein degradation pathways (such as UBQLN2) that lead to proteostasis dysfunction. The link between protein degradation pathways and ALS and FTD is additionally supported by the key pathological hallmark of insoluble proteinaceous inclusions of ubiquitylated proteins, including TDP-43 [14].

We have recently identified ALS–FTD linked mutations in the *CCNF* gene that occur at similar frequency to mutations in *TARDBP* [15]. A p.Ser621Gly (S621G) mutation was found to segregate across multiple generations in an Australian ALS/FTD family, and mutations in other sites across the protein were identified in ALS/FTD patients from the USA, Europe and Japan. Experimental expression of *CCNF* mutations identified from ALS/FTD patients led to defective protein degradation and signature features of ALS pathogenesis *in vitro* including elevated ubiquitylation and increased levels of ubiquitylated ribonucleotide reductase M2 (RRM2) and TDP-43 [15].

Cyclin F is a part of a Skp1-Cul-F-box (SCF) E3 ubiquitin-protein ligase complex which is an integral part of a cell's recycling system that ubiquitylates substrates for degradation by the proteasome. Ubiquitylation is carried out in three main steps: activation by E1s, ubiquitin conjugation by E2s and substrate ligation by E3s. This sequential cascade is the tethering of ubiquitin to lysine residues on proteins via different ubiquitin linkages (such as lysine 48 (Lys48) or lysine 63 (Lys63)), which in turn directs the tagged protein for various purposes including signalling and degradation.

There are over 300 known E3 ligases [16], and it has become increasingly apparent that the activity and substrate selectivity of many E3 ligases is regulated by other post-translational modifications, including phosphorylation [17–20], indicating a convergence of molecular and signalling pathways. For example, phosphorylation of the C-terminus of mouse double minute 2 homolog (MDM2) by the kinase ataxia telangiectasia mutated (ATM) allosterically inhibits really interesting new gene (RING) domain oligomerization resulting in reduced ubiquitylation and stabilization of p53 after DNA damage [20]. Another example is the phosphorylation of Smurf1 at Thr306 by protein kinase A, which regulates its substrate preference during axonal development by preventing degradation of Par6 and increasing degradation of growth-inhibiting RhoA [19]. In the context of ALS and FTD, it was recently found that phosphorylation of TDP-43 by truncated casein kinase (CK) 1δ triggers mislocalization and accumulation of TDP-43 within insoluble aggregates [21], which are the pathological hallmarks observed in most ALS and FTD patients [22]. Numerous kinases have been implicated in TDP-43 phosphorylation including CK1, CK1ɛ, CK2, CDC7 and TTBK1/2 [23–27].

In this study, we investigated the phosphorylation status of cyclin F and the effect of the S621G mutation on its E3 ligase Lys48-specific ubiquitylation activity. We used a phosphoproteomics workflow to identify several novel phosphorylation sites within cyclin F, and identified a key role of CK2 phosphorylation on Ser621 in regulation of SCF^(cyclin F)^ E3 ligase activity. These findings provide insights into the molecular mechanisms of a newly discovered ALS/FTD gene *CCNF*, suggesting that the cyclin F^S621G^ mutation affects Lys48-ubiquitylation activity, with potential consequences for protein degradation pathways that may drive disease development. The convergence of phosphorylation and disease-linked mutation of a single residue (S621G) indicates that this site is of particular importance for ALS/FTD.

## 2. Material and methods

### 2.1. Reagents

Antibodies used in this study were: rabbit polyclonal anti-cyclin F (1 : 300; cat# sc-952, Santa Cruz Biotechnology), mouse monoclonal anti-mCherry (1 : 300; cat# 632543, Clonetech), rabbit anti-mCherry (cat# ab183628, Abcam), rabbit anti-Skp1 p19 (H-163) (1 : 1000, cat# sc-7163, Santa Cruz Biotechnology), mouse monoclonal anti-β-tubulin (1 : 2000; cat# T0198, Sigma-Aldrich) and anti-phospho-CK2 substrate (1 : 1000, cat# 8783, Cell Signaling Technology). Rabbit antibodies against phospho-cyclin F (immunogen: DQEpSEGEKEG) and pan-cyclin F (immunogen: DQESEGEKEG) were customized and purchased from Bethyl Laboratories. Recombinant casein kinase II was obtained from New England BioLabs. Plasmid DNA constructs were purchased from Genscript (Piscataway, NJ, USA)

### 2.2. Cell culture and transfection

Human embryonic kidney (HEK293) or Neuro-2a cell lines were plated at 1 × 10^6^ cells in 100 mm plates and maintained in Dulbecco's modified Eagle's medium (DMEM; Sigma-Aldrich) supplemented with 10% heat-inactivated fetal bovine serum (FBS; Sigma-Aldrich) and 1% antibiotics (100 mg ml^−1^ streptomycin and 100 U mL^−1^ penicillin; Sigma-Aldrich) in a 37°C heat-jacket humidified incubator with 5% CO_2_. Following 48 h of growth (approx. 80% confluence), HEK293 or Neuro-2a cells were transfected with DNA constructs as previously described [15]. Briefly, 7.5 µg of wild-type or mutant cyclin F^S621G^ cDNA fused to N-terminal mCherry was mixed with Lipofectamine 2000 (Life Technologies) and Opti-MEM (Life Technologies) for each plate and incubated at 37°C for 5 h. Cells were washed with warm PBS, re-fed with complete DMEM media and maintained for 24 h. Transfections were carried out in three biological replicates from different passages. For CK2 inhibition, cells were re-fed with complete DMEM media and treated for 18 h with CX4945 (4 µM) prior to harvesting. For CK2 RNAi knockdowns, 25 pmol of CK2α siRNA I (Cat #6389, Cell Signaling Technology, MA, USA) was co-transfected under the same conditions as with mCherry–cyclin F.

### 2.3. Cell lysis and immunoprecipitation

Cells were lysed and total protein was extracted with probe sonication (10 s, Setting 3, Branson Sonifier 450) in NP40 (1% (v/v) Nonidet P-40 in TBS (50 mm Tris-HCl, pH 7.5, 150 mm NaCl) or RIPA (1% (v/v) Nonidet P-40, 0.5% (w/v) sodium deoxycholate, 0.1% (w/v) SDS in TBS) buffer containing 2 mM EDTA, 10 mM N-ethylmaleimide, Complete protease inhibitor cocktail (Roche) and PhosStop inhibitor cocktail (Roche). Cellular debris was pelleted at 16 000*g* (30 min at 4°C). Protein concentration was estimated using the Pierce BCA Reagent (Pierce Biotechnology). Typically, 1 µg of antibody per 500 µg of protein extract in NP40 buffer was used for immunoprecipitations. Protein A/G magnetic beads (Pierce) were used to capture the antibody : protein complex. Immunoprecipitation mixtures were washed with TBS containing 1% (v/v) NP40 (3×) to remove non-specifically bound proteins, and then resuspended in either (i) E3 ligase reaction buffer or (ii) 100 mM ammonium bicarbonate pH 8.0 for in-solution trypsin digestion and LC–MS/MS analyses. Triplicate immunoprecipitations were carried out from three biological transfected cell culture replicates.

### 2.4. One-dimensional SDS-PAGE and immunoblotting analyses

For one-dimensional SDS-PAGE, denatured proteins (20 µg) were separated on 4–12% Bis–Tris pre-cast gels using a 3-(*N*-morpholino) propane sulfonic acid (MOPS) running buffer (180 V, 125 mA) according to the manufacturer's instructions (Invitrogen, MO, USA). One-dimensional SDS-PAGE separated proteins were transferred onto either nitrocellulose or PVDF membranes using the Bio-Rad Turbo Transfer apparatus (13 V, 1.3 A for 7 min for mini-gels or 25 V, 2.5 A for 10 min for midi-gels). Blots were blocked with 3% (w/v) skim milk in TBS/T for 1 h and washed in TBS/T (3×). Primary antibodies were diluted in 3% (w/v) BSA in TBS/T and incubated with blots overnight at 4°C.

After incubation, membranes were washed in PBS/T (3×) for 10 min each before fluorescently labelled IRDye 800CW goat anti-rabbit IgG (1 : 10 000) was applied for 1 h at room temperature. Proteins were imaged using a Li-Cor Odyssey imaging system at the appropriate wavelength. Densitometry analysis was conducted using ImageJ software (v. 1.47; National Institutes of Health) [28] and statistics were conducted using Microsoft Excel. Graphs were made using GraphPad Prism 5 and Microsoft Excel.

### 2.5. *In vitro* phosphorylation by casein kinase II

*In vitro* phosphorylation of naked DQESEGEKEG peptide (Synpeptide, Shanghai, China) was carried out according to the manufacturer's instructions (New England BioLabs, MA, USA). Briefly, the naked peptide (0.2 mg) was resuspended in 180.6 µl of MilliQ water (1 nmol µl^−1^). Approximately 0.1 nmol of peptide was added to the reaction buffer (50 mM Tris-HCl, 10 mM MgCl_2_, 0.1 mM EDTA, 2 mM DTT, 0.01% Brij 35, pH 7.5) with recombinant CK2 (250 U) and 400 µM ATP. The *in vitro* phosphorylation assay was carried out in triplicate and incubated at 30°C for 2 h. Inhibition of CK2 phosphorylation was carried out in the presence of CX4945 (4 µM). The reaction mixture was stopped by addition of 2% (v/v) formic acid, desalted using a C_18_ Sep-Pak and eluted in 90% ACN, 0.1% formic acid and dried under vacuum centrifuge.

### 2.6. Trypsin, Asp-N digestion and titanium dioxide (TiO_2_) enrichment

Immunoprecipitated proteins were reduced and alkylated with 10 mM DTT and 55 mM iodoacetamide respectively, and digested with trypsin or Asp-N (1 : 50 enzyme : protein) overnight at 37°C. The digestion was inactivated by the addition of 2 µl of formic acid (final concentration 2% (v/v) formic acid). Tryptic or Asp-N digested peptides were cleaned and enriched on a pre-equilibrated C_18_ Sep-Pak cartridge and eluted in 90% ACN, 0.1% formic acid and dried under vacuum centrifugation.

Lyophilized peptides were resuspended in 80 mg ml^−1^ glycolic acid, 80% (v/v) ACN, 5% (v/v) TFA and added to TiO_2_ beads (5 mg; GL Sciences, Japan) prewashed in the same solution [29]. The mixture was incubated for 1 h at room temperature with mixing at 1400 r.p.m., and centrifuged at 800*g* to recover the beads. The supernatant was removed and re-added to a fresh batch of TiO_2_ beads and repeated. TiO_2_ beads from both incubations were washed 2× in 80 mg ml^−1^ glycolic acid, 80% (v/v) ACN, 5% (v/v) TFA, 2× in 80% (v/v) ACN, 1% (v/v) TFA, and 1× in 20% (v/v) ACN, 0.1% (v/v) TFA. The beads were briefly dried under vacuum centrifuge. Phosphopeptides were eluted sequentially with 1% NH_4_OH, 1% NH_4_OH/30% ACN and 1% NH_4_OH/50% ACN. The combined eluates were pooled and lyophilized under vacuum centrifugation.

### 2.7. LC–MS/MS

Peptide fractions were injected onto a 2 cm desalting trap column packed with YMC C_18_ material (75 µm ID, 5–15 µm, 120 A) at 5 µl min^−1^ for 6 min before being eluted onto an analytical column packed with Michrom Magic C_18_ (75 µm × 15 cm, 5 µm, 120 A) using a nanoLC system (Thermo) with a nanoflow solvent delivery of 300 nl min^−1^. Each sample was separated on a 60 min gradient (2%–50% v/v acetonitrile, 0.1% formic acid) with a flow rate of 300 nl min^−1^. The peptides were eluted and ionized into a Q-Exactive or LTQ Orbitrap Velos mass spectrometer (Thermo Fisher). The electrospray source was fitted with an emitter tip 10 µm (New Objective, Woburn, MA) and maintained at 2.5 kV electrospray voltage. Precursor ions were selected for MS/MS fragmentation using a data-dependent ‘Top 10’ method operating in FTMS acquisition mode with HCD (Q-Exactive) or CID (Orbitrap) fragmentation. Precursor ions were selected for MS/MS fragmentation using a data-dependent ‘Top 10’ method operating in FT–FT acquisition mode with CID or HCD fragmentation. IT-MS and FT-MS on the Orbitrap Velos was carried out with a survey scan range between *m/z* 350–1800 Da with MS/MS threshold of 500 ions for CID, and MS/MS threshold of 5000 ions for HCD, with an isolation width of 2.0 Da and normalized collision energy of 35%.

FT-MS analysis on the Q-Exactive was carried out with a 35 000 resolution and an AGC target of 1 × 10^6^ ions in full MS; and MS/MS scans were carried out at 17 500 resolution with an AGC target of 2 × 10^5^ ions. Maximum injection times were set to 120 and 60 ms, respectively. The ion selection threshold for triggering MS/MS fragmentation was set to 25 000 counts and an isolation width of 1.9 Da was used to perform HCD fragmentation with normalized collision energy of 27%.

Spectra files (*.RAW) were processed using the Proteome Discoverer 1.4 software (Thermo Finnigan, CA, USA) incorporating the Mascot search algorithm (Matrix Sciences, UK). Peptide identifications were determined using a 10 ppm precursor ion tolerance and a 0.05 Da MS/MS fragment ion tolerance. Carbamidomethylation modification of cysteines was considered a static modification while oxidation of methionine, acetyl modification on N-terminal residues and phosphate on serine, threonine and tyrosine were set as variable modifications. MS/MS spectra were searched through Proteome Discoverer (Thermo Scientific) using the Mascot algorithm against the UniProt *Homo sapiens* database (sequences 15742024, 7 May 2012). The protein sequence for the mutated cyclin F^S621G^ was added manually to a custom database which included the UniProt sequences. The data were processed through the Xtract and MS2 Processor nodes together with a direct search and the combined searches were sent to Percolator (Department of Genome Sciences, University of Washington) for estimation of false discovery rates. Protein identifications were validated employing a *q*-value of 0.01 (1% false-discovery rate) within the Proteome Discoverer software. Phosphosite occupancy was validated using the PhosphoRS 3.1 node within the PD 1.4 with a fragment mass tolerance of 0.5 Da [30]. The data has been deposited to the ProteomeXchange with identifier PXD004531.

### 2.8. E3 ligase activity assay

E3 ligase activity was determined using the E3LITE Customisable Ubiquitin Ligase ELISA Kit (LifeSensors, PA, USA) as per the manufacturer's instructions. UBE2D3 was selected as the E2 enzyme and Lys48-ubiqitin was selected as the ubiquitin substrate. Briefly, empty mCherry vector, mCherry–cyclin F or pre-immune rabbit control IgG were immunoprecipitated from transfected HEK293 cells as described above. Protein A/G beads were washed (3×) in 100 mM Tris-HCl (pH 8.0), 10 mM MgCl_2_ and resuspended in 25 µl assay buffer (100 mM Tris-HCl, 10 mM MgCl_2_, 0.2 mM DTT, pH 8.0). Immunoprecipitated mCherry, mCherry–cyclin F^WT^, mCherry–cyclin F^S621G^ and pre-immune rabbit control IgG were then added to wells containing the Enzyme cocktail (E1 activating enzyme, E2 conjugating enzyme UBE2D3 and recombinant Lys48-ubiquitin). Each ligase reaction was activated by the addition of ATP (0.4 mM) and incubated at RT for 30 min. Each well was washed with PBS/T (3×), incubated with detection solution 1 for 60 min at RT, followed by incubation with streptavidin–HRP for 60 min at RT. Luminescence was read on a FLUOstar OPTIMA reader (BMG Labtech).

## 3. Results and discussion

In this study, we identified that Ser621 on SCF^(cyclin F)^ was phosphorylated and required for regulating its Lys48-specific E3 ligase activity. We determined that phosphorylation at Ser621 is carried out by CK2, and inhibition of CK2 with CX4925 blocks phosphorylation at this site and subsequently elevates SCF^(cyclin F)^ E3 ligase activity. Further, a phosphomimetic created by mimicking constitutive phosphorylation of Ser621 through mutation to an aspartic acid (S621D) led to reduction in the ubiquitylation activity of cyclin F. Therefore, phosphorylation at Ser621 appears to act as a signal for cyclin F to suppress its E3 ligase activity, which can be regulated by controlling CK2 kinase activity or CK2 gene expression. Notably, we demonstrate that a recently identified S621G mutation in cyclin F which causes ALS/FTD [15] also leads to overactivity in the ubiquitylation activity of cyclin F, because Ser621 cannot be phosphorylated. The significance of this finding is that it suggests that abnormal phosphorylation of Ser621 in cyclin F may perturb cellular homeostasis leading to abnormal hyperubiquitylation of proteins, contributing to the aetiology of ALS/FTD.

### 3.1. The ubiquitylation E3 ligase activity of cyclin F is regulated by phosphorylation

**Figure 1.**
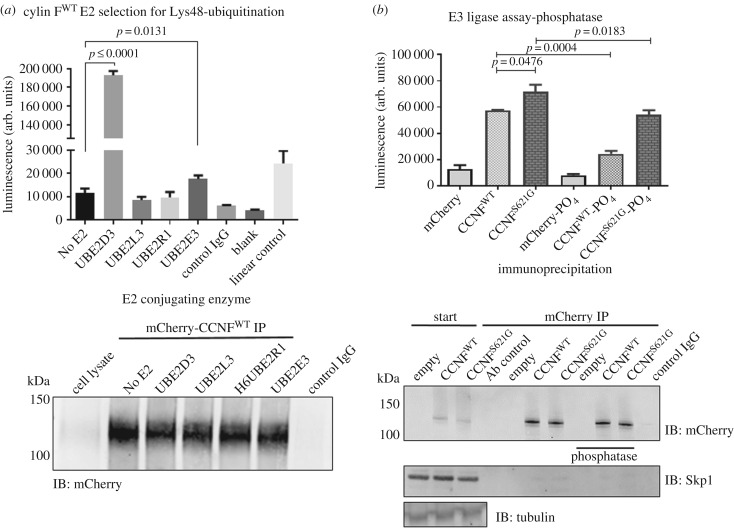
SCF^(cyclin F)^ E3 ligase activity is regulated by phosphorylation. E3 ligase activity assays were used to assess the most suitable E2 conjugating enzyme for mCherry–cyclin F to ubiquitylate substrates. (*a*) Comparison of four common E2 enzymes (UBE2D3, UBE2L3, UBE2R1 and UBE2E3) found that UBE2D3 was the most suited and provided the highest conjugation activity for SCF^(cyclin F)^ (*p* ≤ 0.0001, *n* = 3, Student's *t*-test) followed by UBE2E3 (*p* *=* 0.0131, *n* = 3). (*b*) SCF^(cyclin F)^ was immunoprecipitated from transfected HEK293 cells, treated with Antarctic phosphatase and Lys48-specific E3 ligase activity was measured. The E3 ligase activity of cyclin F^S621G^ appeared to be more active than the wild-type by approximately 1.3-fold (*n* = 3, *p* *=* 0.0476, one-way ANOVA), and following dephosphorylation the E3 ligase activity of immunoprecipitated SCF^(cyclin F)^ was significantly reduced in both mutant and wild-type cyclin F respectively by 1.3-fold (*p* *=* 0.0183, *n* = 3, one-way ANOVA) and 2.3-fold (*p* *=* 0.0004, *n* = 3, one-way ANOVA). Data are represented as the mean ±s.e.m. using one-way ANOVA with Tukey's *post hoc* test. Immunoblots were carried out following E3 ligase activity assays to demonstrate equivalent levels of immunoprecipitated mCherry–cyclin F used for activity assays.

The SCF^(cyclin F)^ complex is one of the many E3 ligases that are responsible for ubiquitylating proteins for UPS degradation. Using a commercial ELISA, we screened for four different E2 conjugating enzymes (UBE2D3, UBE2L3, UBE2R1, UBE2E3) that promiscuously select a large range of substrates [31] (figure 1*a*). Neither UBE2L3 nor UBE2R1 showed Lys48-ubiquitylation activity, suggesting that these two E2s did not form the E1–E2–E3 triad with the immunoprecipitated SCF^(cyclin F)^ complex. UBE2E3 showed E2 Lys48-conjugation activity approximately twofold above the background (no E2) (*p* *=* 0.0131, *n* = 3). UBE2D3 showed high Lys48-specific ubiquitylation activity and was approximately eightfold higher than UBE2E3 and 16-fold higher than the background controls (*p* ≤ 0.0001, *n* = 3). Based on these data, UBE2D3 was selected as the E2 conjugating enzyme to be used in downstream E3 ligase activity assays. Immunoblots were carried out following E3 ligase activity assays to demonstrate equivalent levels of immunoprecipitated mCherry–cyclin F used for activity assays (figure 1*a*).

To determine the E3 ligase activity, wild-type and ALS/FTD mutant cyclin F^S621G^ were immunoprecipitated from transfected HEK293 cells, treated with Antarctic phosphatase to remove phosphate, and the Lys48-specific E3 ligase activity was measured on bead by ELISA. The E3 ligase activity of mutant cyclin F^S621G^ was approximately 1.3-fold more active than wild-type cyclin F (*p* *=* 0.0476, *n* = 3). Given that serine residues are well-described phosphosites that often regulate enzymatic activity, this suggests that the phosphorylation of the Ser621 site might regulate ubiquitylation activity of cyclin F (and, therefore, loss of this phosphosite through the S621G mutant increases ubiquitylation activity). Accordingly, following complete dephosphorylation the E3 ligase activity of immunoprecipitated SCF^(cyclin F)^ was significantly decreased in both mutant and wild-type cyclin F (figure 1*b*). This effect was greater in the wild-type (2.3-fold, *p* *=* 0.0004, *n* = 3) than the mutant cyclin F^S621G^ (1.3-fold, *p* *=* 0.0183, *n* = 3). This led us to speculate that the activity of the SCF^(cyclin F)^ complex was regulated by phosphorylation of multiple sites, including Ser621, which would explain the difference seen in the decreased activity of wild-type compared with mutant cyclin F^S621G^ following dephosphorylation. Immunoblots were carried out following E3 ligase activity assays and demonstrated that the ubiquitylation activity measurements were performed on equivalent levels of immunoprecipitated mCherry–cyclin F (figure 1*b*).

### 3.2. Identification of phosphosites in cyclin F

The *in vitro* ubiquitylation assays (figure 1) suggest that Ser621 might be a phosphorylation site that regulates about 30% of the ubiquitylation activity of cyclin F, which was confirmed by predictions using NetPhos 2.0 (http://www.cbs.dtu.dk/services/NetPhos/). NetPhos predicted with high confidence 88 potential phosphorylation sites in cyclin F, which included Ser621. To determine phosphorylation sites on cyclin F, transfected mCherry–cyclin F was enriched by immunoprecipitation, digested with trypsin, phosphopeptides enriched by TiO_2_ beads, and analysed by LC–MS/MS. Using this digestion procedure, we identified two phosphorylation sites on cyclin F at Ser577 and Ser754 (table 1). The regions surrounding Ser621 do not contain lysine or arginine residues that are required for producing appropriate length tryptic peptides for LC–MS. Instead, we employed Asp-N protease to digest cyclin F, enriched Asp-N digested peptides using TiO_2_ and subjected them to LC–MS/MS. Using this strategy we observed specific diagnostic ions and loss of phosphate ions (PO_4_) to detect an additional four phosphosites on cyclin F: Thr588, Thr590, Ser621, Ser709 and Ser713 [32] (table 1).

**Table 1.** Cyclin F phosphorylation sites. Two digestion strategies: trypsin and Asp-N were used to digest immunoprecipitated mCherry–cyclin F from transfected HEK293 or Neuro-2a cells. Seven phosphorylation sites were identified, of which five have not been reported and are located within the PEST sequence of cyclin F. Group-based prediction system (GPS) was used to predict upstream kinases of each phosphorylation site identified.

phosphopeptide sequence	start	end	site	predicted upstream kinase
ENpSLQEDR (trypsin)	575	582	S577^a^	AKT
DRGSFVTpTPTAELSSQEETLLGSFL (Asp-N)	581	605	T588^a^	CAMK
DRGSFVTTPpTAELSSQEETLLGSFL (Asp-N)	581	605	T590^a^	CAMK
DQEpSEGEKEG (Asp-N)	618	627	S621^a^	CK2
DVTTSGYSSVSTApSPTSSV (Asp-N)	696	714	S709^a^	P38 MAPK
DVTTSGYSSVSTASPTSpSV (Asp-N)	696	714	S713	P38 MAPK
SCLQCRPPpSPPESSVPQQQVK (trypsin)	745	766	S754	CDK4/5

^a^Unique phosphorylation site.

**Figure 2.**
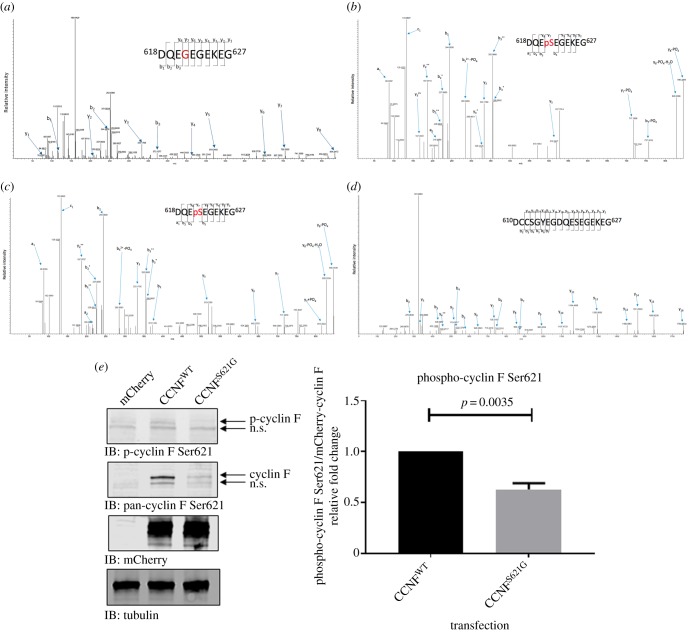
Cyclin F Ser621 is phosphorylated. mCherry–cyclin F from transfected HEK293 cells was immunoprecipitated for phosphorylation analysis (*a*) MS/MS spectra of mutated Asp-N digested cyclin F peptide ^618^DQEGEGEKEG^627^; (*b*) MS/MS spectra of Asp-N digested wild-type cyclin F peptide ^618^DQEpSEGEKEG^627^; (*c*) MS/MS spectra of synthetic cyclin F peptide DQEpSEGEKEG showing almost identical b- and y-ions to the spectra shown in (*b*); (*d*) MS/MS spectra of phosphatase treated wild-type cyclin F, followed by Asp-N digestion showing complete removal of phosphate prior to MS analysis; and (*e*) cyclin F^WT^ and cyclin F^S621G^ were immunoblotted using custom rabbit antibodies raised against DQEpSEGEKEG (phospho) and DQESEGEKEG (pan) confirming the phosphorylation status at Ser621 and reduced phosphorylation by 0.37-fold (*p* *=* 0.0035, *n* = 3, Student's *t*-test).

The phosphorylation status of immunoprecipitated mCherry–cyclin F containing the S621G mutation (figure 2*a*) and wild-type mCherry–cyclin F^WT^ (figure 2*b*) was confirmed by MS/MS fragmentation. To verify this phosphorylated sequence, we subjected a synthetic phosphorylated (figure 2*c*) and unphosphorylated (not shown) versions of the Asp-N digested peptide (DQESEGEKEG) to LC–MS/MS. We confirmed the MS/MS fragmentation spectra matched between both synthetic and immunoprecipitated peptides. We also immunoprecipitated mCherry–cyclin F and dephosphorylated the IP prior to LC–MS/MS analysis and observed MS/MS fragmentation patterns that were reminiscent of the unphosphorylated version of the peptide (figure 2*d*), further confirming that Ser621 is a phosphorylation site. The MS/MS spectra that confirm the phosphosites described in table 1 are available on the ProteomeXchange with identifier PXD004531.

Using an alternative method, we examined the phosphorylation of cyclin F by immunoblots using customized antibodies raised against the phosphopeptide sequence DQEpSEGEKEG and non-phosphorylated DQESEGEKEG (Bethyl Laboratories). Although there was very minor non-specific reactivity observed with the phospho-specific cyclin F antibody, we verified that cells expressing only mCherry–cyclin F^WT^ were phosphorylated at Ser621 with negligible amounts detected in the mutant cyclin F^S621G^ and empty vector transfected cells (figure 2*e*). We detected reduced phosphorylation by approximately 0.37-fold (*p* *=* 0.0035, *n* = 3) of phospho-specific signal in cyclin F^S621G^ mutant cell lysates compared with cyclin F^WT^ lysates (figure 2*e*). We predicted that the non-phospho-specific antibody would detect less of the mutant cyclin F^S621G^ than the wild-type cyclin F, and confirmed that the antibody reactivity observed was indeed cyclin F. These data confirm the mass spectrometric identification of phosphorylated cyclin F at Ser621. Taken together, using both trypsin and Asp-N digestion approaches seven phosphorylation sites were identified, of which five have not been reported previously (S577, T588, T590, S621, S709) and two have been previously characterized (S713, S754) [32,33] (table 1). The phospho-specific antibody to cyclin F was able to distinguish between phosphorylated wild-type and mutant cyclin F, and therefore we used this antibody for further validation to determine the relative level of cyclin F phosphorylation upon cell treatments.

### 3.3. Casein kinase II phosphorylates cyclin F at Ser621

Many SCF complexes function by ubiquitylating substrates in a phosphorylation-dependent manner [34] such that the cyclin-dependent kinase (CDK) catalytic subunits are used to phosphorylate substrates and the F-box protein acts as a receptor to recruit phosphorylated substrates to the complex [35]. For example, phosphorylated cyclin D1 (Thr286) is ubiquitylated by SCF^(FBX4-alphaB crystallin)^ prior to proteolysis and is required for regulating cell cycle progression such that impairment of SCF^(FBX4-alphaB crystallin)^ function attenuates cyclin D1 ubiquitylation, promoting cyclin D1 overexpression and accelerated cell-cycle progression [36]. Cyclin F does not bind CDKs and instead a hydrophobic patch within its cyclin box domain binds a CY motif (RxL/RxI) in the substrate [37]. Therefore, cyclin F is not phosphorylated by the canonical series of cyclin-dependent kinases and must be modified by kinases that are independent of cell-cycle regulation to ultimately regulate the SCF^(cyclin F)^ complex. Using the group-based prediction system (GPS) [38], numerous MAPKs, CDKs and NEKs were identified as potential kinases that could phosphorylate the newly identified phosphosites in cyclin F (table 1).

**Figure 3.**
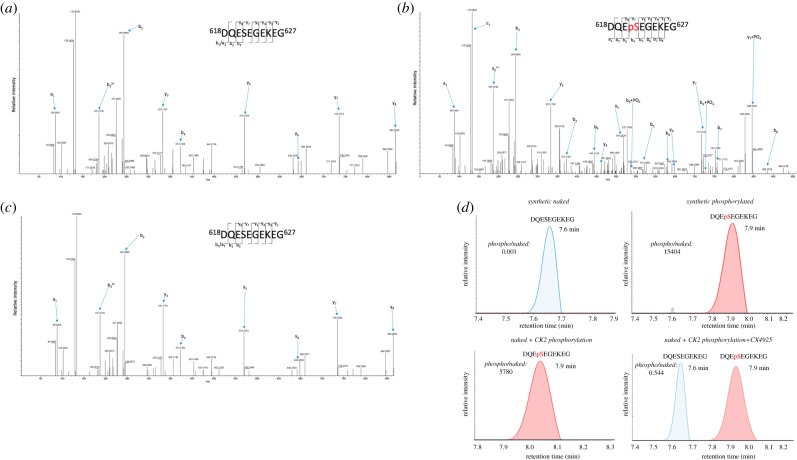
Casein kinase II is responsible for phosphorylating Ser621 on cyclin F. (*a*) MS/MS spectra of synthetic unphosphorylated DQESEGEKEG peptide; (*b*) MS/MS spectra of *in vitro* phosphorylation of synthetic DQEpSEGEKEG peptide by recombinant CK2; (*c*) MS/MS spectra of *in vitro* phosphorylation of synthetic DQESEGEKEG peptide by recombinant CK2 in the presence of 4 µM CX4945 inhibitor; (*d*) EIC and matching elution times of *m/z* 554.2 (unphosphorylated) at 7.6 min and 594.2 (phosphorylated) DQESEGEKEG peptide at 7.9 min demonstrates that both peptides have distinct elution times and provides additional confidence of the existence of the identification of phosphorylated cyclin F at Ser621.

Casein kinase II (CK2) was predicted to phosphorylate Ser621 with the surrounding sequences falling within the CK2 substrate motif (S–X–X–D/E). Using the synthetic naked peptide (DQESEGEKEG) which eluted at 7.6 min (figure 3*a*), we performed an *in vitro* phosphorylation assay with recombinant CK2 and ATP. The reaction was analysed by LC–MS/MS, which revealed phosphorylation on serine within the synthetic peptide that corresponds to Ser621 in cyclin F that eluted at 7.9 min (figure 3*b*). We repeated the *in vitro* phosphorylation assay in the presence of the CK2 inhibitor CX4945 and analysed the reaction by LC–MS/MS (figure 3*c*). Using extracted ion chromatograms (EICs) and matching retention times of 7.6 and 7.9 min to respectively identify the unphosphorylated and phosphorylated peptides and their specific masses, a mixture of 45% naked DQESEGEKEG peptide (*m/z* 554.2) and 55% phosphorylated DQEpSEGEKEG peptide (*m/z* 594.2) was observed. CX4945 treatment decreased recombinant CK2 phosphorylation activity by approximately 55% based on area-under-curve measurements (figure 3*d*). We suspect that the incomplete inhibition of CK2-mediated phosphorylation of Ser621 by CX4945 was likely caused by the presence of a 100-fold higher concentration of ATP than CX4945 (which was required for the *in vitro* ubiquitylation assay), as CX4945 acts by blocking the ATP-binding site in CK2 (and therefore CX4945 and ATP are competing for the same site in CK2) [39]. Attempts to overcome this by performing the assay with lower concentrations of ATP interfered with the quality of the ubiquitylation assay (results not shown), and so we were unable to confirm this experimentally. These results demonstrate that CK2 can phosphorylate the serine within the synthetic DQESEGEKEG peptide, corresponding to amino acids 618–627 of cyclin F. Combined with the bioinformatics predictions, this provides evidence that CK2 has the ability to phosphorylate Ser621 within cyclin F in cells.

**Figure 4.**
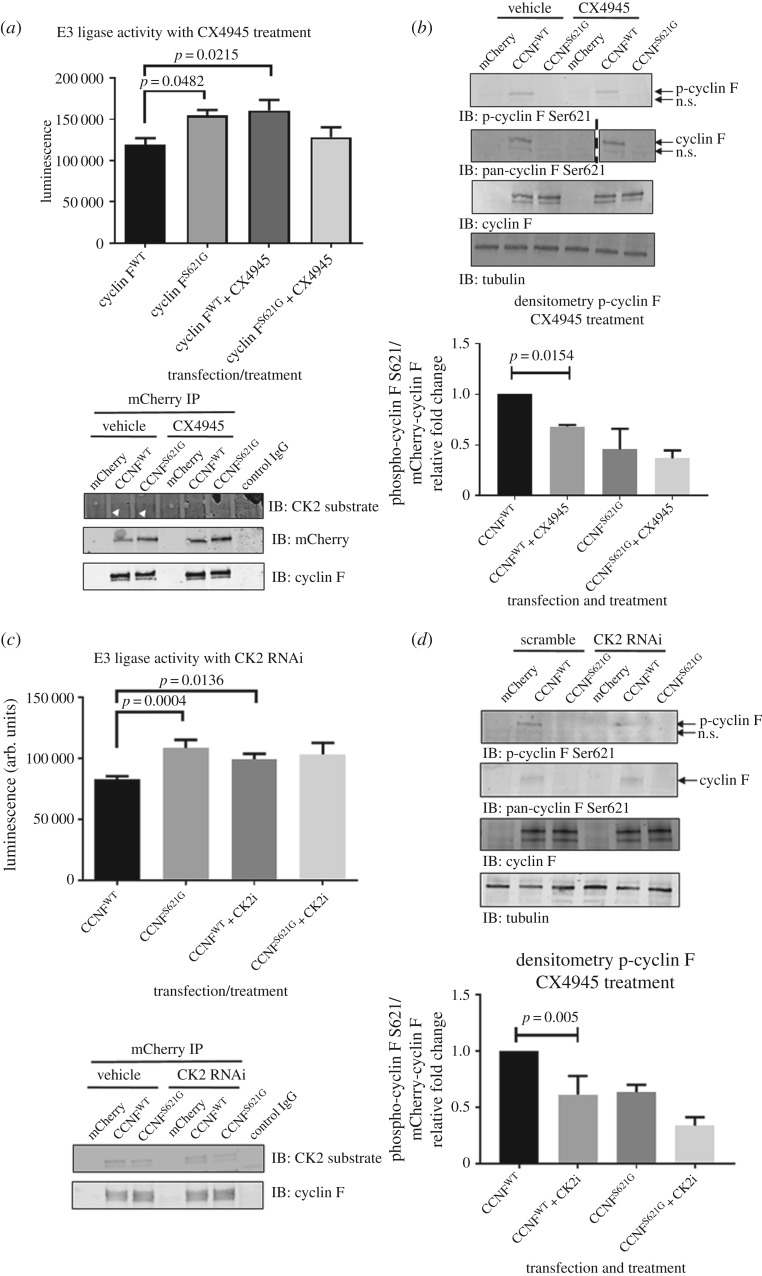
Casein kinase II phosphorylation regulates SCF^(cyclin F)^ Lys48-specific E3 ligase activity. (*a*) E3 ligase activity of Lys48-ubiquitylation from immunoprecipitated mCherry–cyclin F in transfected cells treated with CX4945 (4 µM) demonstrated elevated ubiquitylation activity with CK2 inhibition by approximately 1.35-fold (*p* *=* 0.0215, *n* = 3, one-way ANOVA). Immunoblots were carried out following E3 ligase activity assays to demonstrate equivalent levels of immunoprecipitated mCherry–cyclin F used for activity assays, which also revealed cyclin F contained a CK2 phosphorylation motif. (*b*) Immunoblot analysis of phospho-cyclin F (Ser621) using custom antibodies revealed reduced phosphorylation by approximately 0.68-fold (*p* = 0.0154, *n* = 3, one-way ANOVA) with CX4945 treatment. Vertical dashed line indicates cropped lanes. (*c*) E3 ligase activity of Lys48-ubiquitylation from immunoprecipitated mCherry–cyclin F in cells co-transfected with mCherry–CCNF^WT^ or mCherry–CCNF^S621G^ with scramble or CK2 siRNA demonstrated elevated ubiquitylation activity with CK2 knock down by approximately 1.2-fold (*p* *=* 0.0136, *n* = 3, one-way ANOVA). Immunoblots were carried out following E3 ligase activity assays to demonstrate equivalent levels of immunoprecipitated mCherry–cyclin F used for activity assays. (*d*) Immunoblot analysis of phospho-cyclin F (Ser621) using custom antibodies revealed reduced phosphorylation by approximately 0.61-fold (*p* = 0.005, *n* = 3, one-way ANOVA) with CK2 RNAi knockdown. Data are represented as the mean ± s.e.m. using one-way ANOVA with Tukey's *post hoc* test.

Next, we examined SCF^(CyclinF)^ E3 ligase activity in the presence of CK2 inhibition by transfecting HEK293 cells with cyclin F^WT^ and cyclin F^S621G^ followed by treatment with CX4945 for 18 h. In cells treated with CX4945, immunopurified cyclin F^WT^ had approximately 1.35-fold elevated Lys48-ubqiuitylation activity compared with the vehicle controls (figure 4*a*), while CX4945 treatment did not affect the activity of immunopurified mutant cyclin F^S621G^ based on E3 ligase activity assays. We verified that equivalent levels of immunoprecipitated mCherry–cyclin F were used in the ubiquitylation assay, by probing for mCherry and cyclin F at the completion of the E3 ubiquitylation activity assays (figure 4*a*). We immunoblotted transfected cell lysates treated with CX4945 using the phospho-specific cyclin F (Ser621) antibody, and observed approximately 32% less Ser621 phosphorylation (*p* = 0.0154, *n* = 3) in CX4945 treated cyclin F^WT^ cells (figure 4*b*). To confirm that the CX4945 effectively impairs CK2 activity in these cell-based assays, we performed immunoblotting analyses to detect CK2 phosphorylated substrates containing the S–X–X–D/E motif. Inhibition of CK2 activity by CX4945 resulted in approximately 39% and 37% decreased phosphorylation of substrates in cells transfected with cyclin F^WT^ and cyclin F^S621G^, respectively. The detection of residual CK2 substrates in cells treated with CX4945 is likely due to: (i) endogenous CK2 phosphorylation events that occurred prior to CX4945 treatment because CK2 is constitutively active regulating numerous cellular processes [40] and/or (ii) incomplete inhibition of CK2 phosphorylation activity by CX4945 as described earlier (figure 3*d*).

This finding was recapitulated by siRNA-mediated knockdown of CK2α expression in cells expressing mCherry–cyclin F^WT^ and mCherry–cyclin F^S621G^. In cells expressing cyclin F^WT^, with CK2 knockdown, we observed a statistically significant approximately 20% increase (*p* *=* 0.0136, *n* = 3) in E3 ligase activity (figure 4*c*). We immunoblotted for phospho-specific cyclin F at Ser621 from transfected cell lysates, and observed approximately 0.61-fold less Ser621 phosphorylation (*p* = 0.005, *n* = 3) in CK2 RNAi knockdown cells expressing cyclin F^WT^ compared with those with scramble siRNA control, further suggesting that CK2 is capable of phosphorylating this site (figure 4*d*).

**Figure 5.**
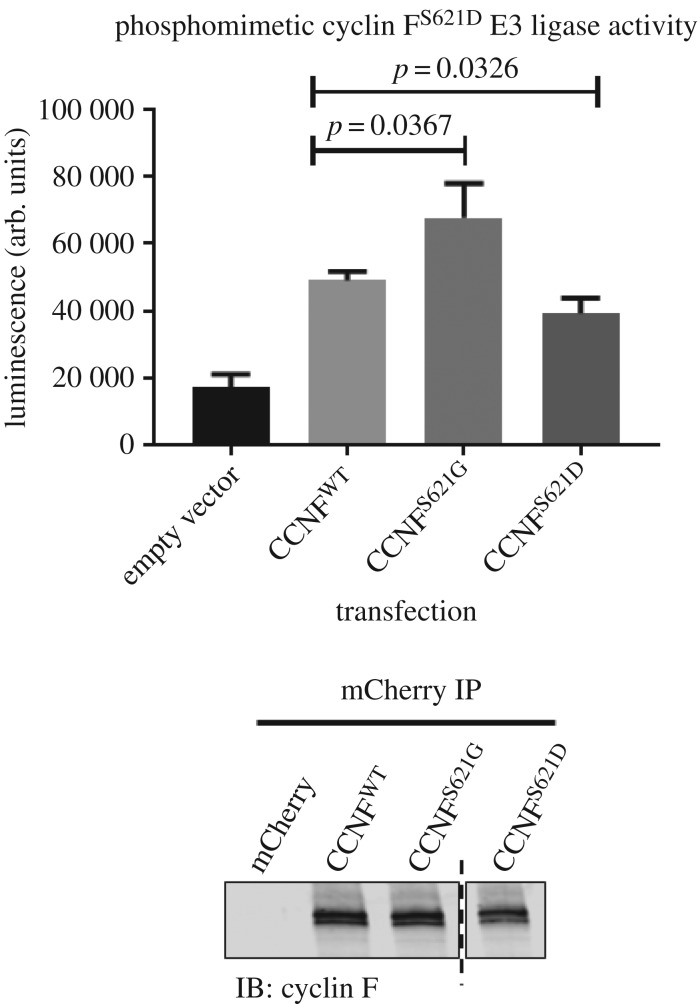
Phosphomimetic cyclin F^S621D^ reduces SCF^(cyclin F)^ Lys48-ubiquitylation activity. E3 ligase activity of immunoprecipitated phosphomimetic mCherry–cyclin F^S621D^ was less active compared with the wild type by approximately 20% (*p* *=* 0.0326, *n* = 3, Student's *t*-test). Immunoblots were carried out following E3 ligase activity assays to demonstrate equivalent levels of immunoprecipitated mCherry–cyclin F used for activity assays. Vertical dashed line indicates cropped lanes.

In this study, we predicted and subsequently confirmed that CK2 phosphorylates Ser621 and, therefore, has a role in regulating the E3 ligase activity of cyclin F. This is important because we have recently identified an ALS/FTD-causing S621G mutation in cyclin F, which presumably prevents phosphorylation of Ser621, resulting in elevated activity of cyclin F^S621G^ that may contribute to the abnormal hyperubiquitylation of proteins, which are a hallmark pathology of ALS and FTD. This suggests that phosphorylation of Ser621 serves to turn off cyclin F activity. To confirm this, we generated a phosphomimetic by mutating Ser621 to an aspartic acid (S621D). As expected, the phosphomimetic cyclin F^S621D^ was approximately 0.8-fold less active than the wild-type cyclin F (*p* *=* 0.0367, *n* = 3) (figure 5). The conversion to an aspartic acid in place of a phosphorylated serine that is chemically similar confers a net negative charge rendering this site as being ‘constitutively phosphorylated’, which resulted in a reduction of E3 ligase activity.

**Figure 6.**
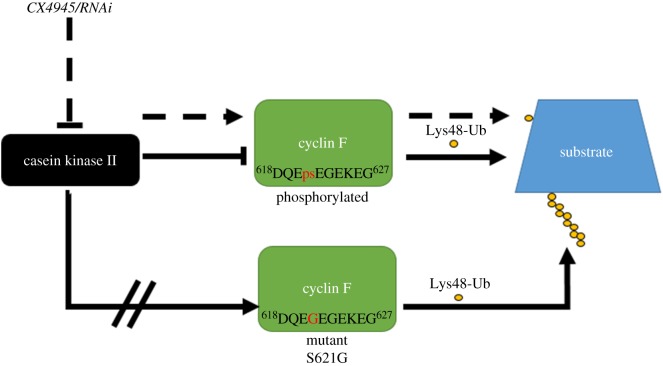
Proposed mechanism of phosphorylated Ser621 in cyclin F and its effect on the SCF^(cyclin F)^ Lys48-ubiquitylation activity. CK2 is responsible for phosphorylating Ser621 in cyclin F and regulating the SCF^(cyclin F)^ E3 ligase activity. Inhibition of CK2 activity by either CX4945 or siRNA knock down prevents phosphorylation of mCherry–cyclin F at Ser621 and elevates its Lys48-specific E3 ligase activity. Additionally, a mutation at Ser621 (p.Ser621Gly) has been found in a large multi-generational Australian family with ALS/FTD, and expression of this mutant version (cyclin F^S621G^) in cells displays elevated Lys48-specific E3 ligase activity.

We present a series of biochemical data that collectively suggest that CK2 phosphorylation of cyclin F at Ser621 is crucial for regulating SCF^(cyclin F)^ complex E3 ligase activity. Notably, the ALS/FTD-causing S621G missense mutation confers elevated E3 ligase activity (figure 6). Our findings suggest that precisely regulated phosphorylation of Ser621 in cyclin F is crucial for the maintenance of appropriate activity of ubiquitylation-dependent protein degradative pathways. These pathways are often impaired in neurodegenerative diseases such as ALS and FTD, indicating that mutation of Ser621 and altered phosphorylation status of cyclin F is likely one of the upstream pathogenic causes of these diseases.

## Supplementary Material

Supplementary Figures

## Supplementary Material

Supplementary Figures

## Data accessibility

Mass spectrometry data has been deposited to the ProteomeXchange with identifier PXD004531 (username: reviewer75905@ebi.ac.uk; password: H2YC2b5w).
