# Supplementary figures and images for: Casein kinase II phosphorylation of cyclin F at serine 621 regulates the Lys48-ubiquitylation E3 ligase activity of the SCF(cyclin F) complex
Source: Open Biol. 2017 Oct 11;7(10):170058. doi: 10.1098/rsob.170058 (PMC5666078; doi:10.1098/rsob.170058)

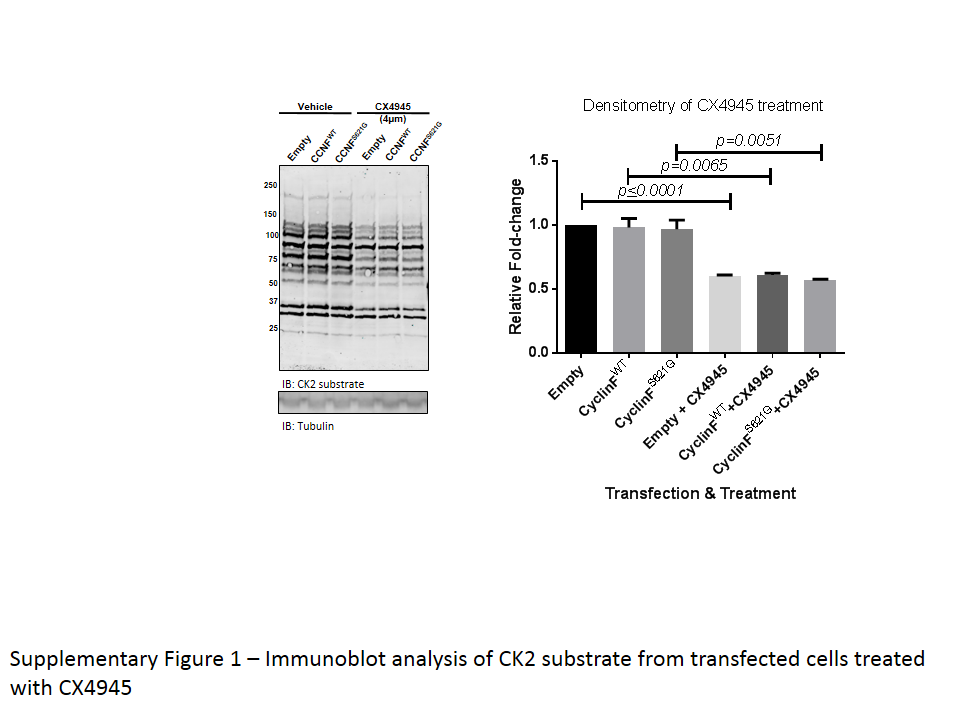

Supplement: Supplementary Figures [file rsob170058supp1.tif]

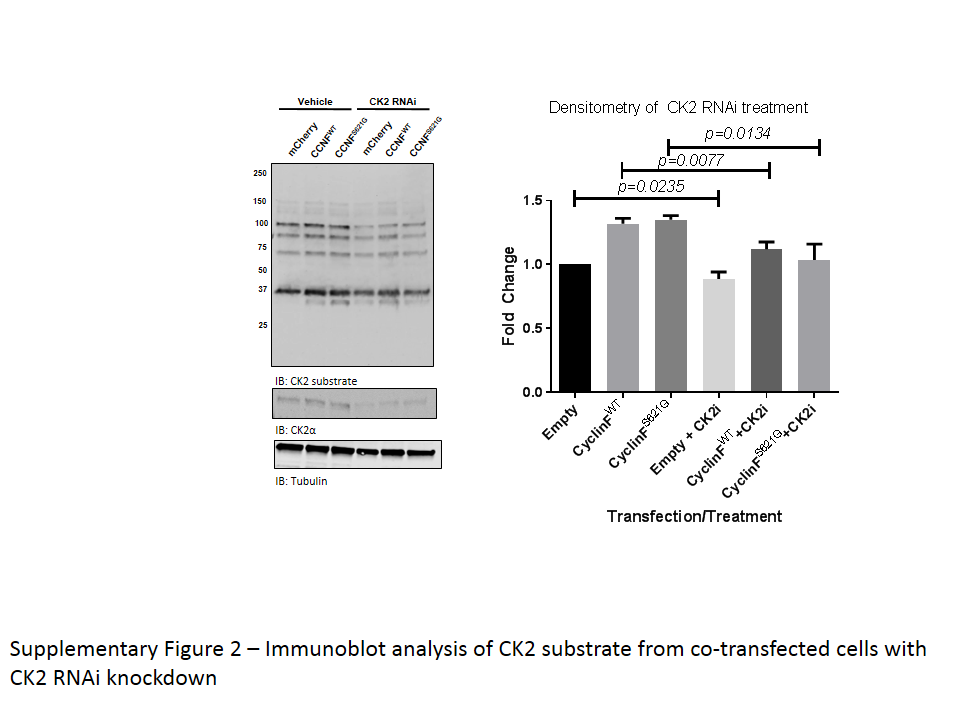

Supplement: Supplementary Figures [file rsob170058supp2.tif]
